# Outcomes of surgical resection for pulmonary metastasis from pancreatic cancer

**DOI:** 10.1007/s00595-023-02701-0

**Published:** 2023-06-14

**Authors:** Yudai Miyashita, Naoko Ose, Jiro Okami, Koji Takami, Yasushi Sakamaki, Naoki Ikeda, Masanobu Hayakawa, Masahiko Higashiyama, Ken Kodama, Yoshiyuki Susaki, Yasunobu Funakoshi, Jun Maeda, Yasushi Shintani

**Affiliations:** 1 Department of General Thoracic Surgery, Osaka University Graduate School of Medicine, 2-15 Yamadaoka, Suita, Osaka 565-0871 Japan; 2 Department of General Thoracic Surgery, Osaka International Cancer Institute, Osaka, Japan; 3 Department of Thoracic Surgery, National Hospital Organization Osaka National Hospital, Osaka, Japan; 4 Department of Chest Surgery, Osaka Police Hospital, Osaka, Japan; 5 Department of Thoracic Surgery, Sakai City Medical Center, Sakai, Osaka Japan; 6 Department of Thoracic Surgery, Moriguchi Keijinkai Hospital, Moriguchi, Osaka Japan; 7 Department of Thoracic Surgery, Higashi Osaka Medical Center, Higashiosaka, Osaka Japan; 8 Department of Thoracic Surgery, Yao Municipal Hospital, Yao, Osaka Japan; 9 Department of Thoracic Surgery, Ikeda City Hospital, Ikeda, Osaka Japan; 10 Department of General Thoracic Surgery, Osaka General Medical Center, Osaka, Japan; 11 Department of Thoracic Surgery, Otemae Hospital, Osaka, Japan

**Keywords:** Pulmonary metastasis, Pancreatic cancer, Pulmonary metastasectomy

## Abstract

**Purpose:**

As the number of long-term survivors of pancreatic cancer is expected to increase thanks to recent advances in multidisciplinary treatment and earlier diagnoses of pancreatic cancer, we are likely to encounter more cases of postoperative pulmonary nodules. We analyzed the clinical course and prognosis of resection of pulmonary metastases from pancreatic cancer to clarify the prognostic implication of pulmonary metastasectomy for pancreatic cancer.

**Method:**

We retrospectively analyzed 35 patients who underwent resection of lung metastases after pancreatic cancer surgery. Short- and long-term outcomes and factors associated with the prognosis were analyzed.

**Results:**

The observation period was 20 (range, 1–101) months, with 3- and 5-year survival rates of 88.3% and 64.5% from pancreatectomy and 44.1% and 28.3% from lung resection, respectively. A univariate analysis revealed that a period from pancreatic cancer resection to pulmonary nodule shadow detection of < 15 months was associated with a significantly lower overall survival from pancreatic resection than a longer period. Conversely, histological type, stage, size of lung metastases, and resection technique were not associated with the overall survival.

**Conclusion:**

A long-term prognosis may be expected in some cases with a disease-free interval of ≥ 15 months. Our findings suggest that the disease-free interval may influence the prognosis.

## Introduction

Pancreatic cancer has one of the poorest prognoses among all types of carcinomas, with a median survival of 12.6 months and a 5-year survival rate of 7% [1]. The poor prognosis of pancreatic cancer is due to the fact that many patients have advanced disease at the diagnosis and experience recurrence after surgery of pancreatic cancer [2, 3]. Even in cases where surgery is performed for pancreatic cancer, the 3-year disease-specific survival rate was reported to be 27% [4].

Morimoto et al. reported that locoregional recurrence was the most common site of recurrence after pancreatic cancer surgery, followed by simultaneous recurrence, liver metastasis, peritoneal dissemination, and lung metastasis, the last of which was reported to be the second-most common distant organ metastasis [5]. Others have reported 6.4–26.7% of patients developing lung metastases after pancreatic cancer surgery [6, 7]. Among the organs of distant metastatic recurrence following pancreatic cancer surgery, metastatic lung recurrence has a later onset and better prognosis than other distant metastases [5, 8, 9]. Zheng et al. reported that the time from initial surgery to recurrence was longer in the lungs than in the liver, peritoneum, or distant lymph nodes; furthermore, the survival after the diagnosis of recurrence was longer for cases involving the lungs than those involving the liver, peritoneum, or distant lymph nodes [8]. Lovecek et al. found that cases of lung metastasis had a longer period from the first surgery to the diagnosis of recurrence than those of lung + other organ metastasis or extrapulmonary metastasis. In addition, lung metastasis alone had a longer overall survival (OS) than lung + other organ metastasis or extrapulmonary metastasis [10]. Kruger et al. showed that patients with ≤ 10 recurrent lung metastases had a significantly longer OS after the diagnosis of lung metastasis than those with ≥ 11 metastases [9].

Of note, recent advances in multidisciplinary treatment and the earlier diagnosis of pancreatic cancer have improved the outcomes of pancreatic cancer [2, 11]. As the number of long-term survivors of pancreatic cancer is expected to increase, we are likely to encounter more cases of postoperative pulmonary nodules in the future.

Pulmonary metastasectomy (PM) is a well-established treatment for several cancer types [12]. It is frequently indicated for solitary lung metastases, and there have been several reports on its application in the treatment of other cancers, including colorectal cancer, renal cell carcinoma, and uterine malignancies; some carcinomas, such as colorectal and renal cell carcinoma, are expected to show an improved prognosis with this approach [12, 13], whereas others, including lung cancer, have been reported to show no marked difference in the prognosis [14]. However, there are few coherent reports on pulmonary resection for postoperative lung metastases from pancreatic cancer.

Given the above, the present study analyzed the clinical course and results of PM for pancreatic cancer in a multicenter setting and clarified the prognostic implication of PM.

## Materials and methods

### Patients

A total of 35 patients who underwent pulmonary resection for postoperative lung nodules from pancreatic cancer at 11 institutions belonging to the Thoracic Surgery Study Group of Osaka University between January 2009 and December 2021 were enrolled (including cases with no preoperative diagnosis). Cases that underwent surgery for biopsy purposes, cases wherein the possibility of lung metastasis from other cancers could not be ruled out due to a history of other cancers, cases wherein enrollment was refused, and cases deemed ineligible by the doctor-in-charge were excluded.

### Pathological diagnoses

Pathologists made the diagnosis of metastatic lung tumors at each institution. Immunostaining (thyroid transcription factor-1 [TTF-1] and napsin A) and genetic testing were performed at the discretion of the diagnosing pathologist. TTF-1-positive cases were determined not to be lung metastases from pancreatic cancer [15, 16]. Cases wherein a histopathological examination could not differentiate between lung metastasis from pancreatic cancer and primary lung cancer were excluded.

### Evaluation criteria

The primary endpoint was the OS, and the secondary endpoint was prognostic factors. The Institutional Review Boards approved the study protocol of the Ethics Committee of Osaka University Hospital (control number 20160–2) and those of the participating hospitals.

### Methods

Pulmonary nodule diagnoses were made based on chest computed tomography (CT) findings. The primary tumor was diagnosed pathologically and treated by surgery in all patients before PM. Tissues of the resected pulmonary nodules were histologically evaluated, and all cases were diagnosed as pulmonary metastasis from pancreatic cancer. Clinical information was collected from the medical records of all participating hospitals.

The indications for pre- and/or postoperative chemotherapy were determined by the general surgeon-in-charge after considering the general condition of the patient and the state of the disease. The type of resection and surgical approach were selected according to the size and location of the recurrent pulmonary metastases. Follow-up was generally based on the findings of chest X-ray or chest CT, a physical examination, and a blood chemistry evaluation, which were performed every 6–12 months after the first PM procedure.

The disease-free interval (DFI) was defined as the interval between treatment for the primary pancreatic tumor and detection of pulmonary metastases and was counted as 0 for patients whose primary and metastatic diseases were simultaneously diagnosed at the initial presentation. The pancreatic OS (P-OS) was defined as the time between the date of pancreas resection and death or last follow-up for surviving patients, and the lung OS (L-OS) was defined as the time between the date of pulmonary resection and death or last follow-up for surviving patients.

In this study, the follow-up period was defined as the interval between the date of pulmonary resection and the date of death or the latest follow-up. In addition, the 75th quartile, median, and 25th quartile follow-up periods were 35, 20, and 11 months, respectively (range, 1–101 months).

### Statistical analyses

The Kaplan–Meier method was used to describe the survival curve and rate. Statistical differences between survival curves were examined using the log-rank test. Cox’s proportional hazards model was utilized to decide the risk factors for recurrence. *P* values < 0.05 were considered statistically significant. Data were expressed as means ± standard deviations or median values. YM (the author) performed all statistical analyses using the JMP pro 16 software program (SAS Institute Inc., Cary, NC, USA).

## Results

### Patient characteristics

The characteristics of the 35 patients are shown in Table 1. Thirty-four cases were characterized by distant metastases solely in the lungs. A single case was identified in which metastasis to the peri-superior mesenteric artery lymph node was present. Chemoradiotherapy was administered, and complete remission was confirmed. Subsequently, lung resection was performed. There were no instances of simultaneous lung and liver metastases.

**Table 1:** Patient characteristics

Factor	
Age, years	
Median(range)	71 (55–90)
Brinkman Index*	
Median (range)	0 (0–3000)
CA19-9, ng/ml*	
Median (range)	19.4 (0–480.6)
CEA, ng/ml	
Median (range)	4.1 (1–35)
Number of lung nodules	
Median (range)	1 (1–13)
Side	
One	27 (77.1)
Both	8 (22.9)
Tumor size, mm	
Median (range)	12 (6–28)
Distance from pleural surface, mm	
Median (range)	1.0 (0–30)
Localization	
LUL	6
LLL	8
RUL	3
RML	3
RLL	15
Border	
Regular	12
Irregular	23
Shape	
Circle, oval	28
Others	7
Cavity	
( +)	6
(−)	29
Calcification	
( +)	0
(−)	35
Consolidation-Tumor ratio	
Median(range)	1.0 (0.33–1.0)
Induction therapy for PM	
( +)	12
(−)	23
Months from detection to PM	
Median (range)	5 (0–35)
Approach	
VATS	31
Thoracotomy	4
Operation	
Wedge	25
Segmentectomy	3
Lobectomy	7
Lymph node dissection	
ND0	24
ND1	5
ND2	3
Unknown	3
Complete resection	
( +)	30
(−)	5
Postoperative hospital stay, days	
Median (range)	9 (4–26)
Immunostaining	
NapsinA	
( ±)	1
(-)	21
Unknown	13
Chemotherapy after PM	
( +)	13
(−)	22

*CEA* carcinoembryonic antigen, *CA19-9* carbohydrate antigen19-9, *LUL* left upper lobe, *LLL* left lower lobe, *RUL* right upper lobe, *RML* right middle lobe, *RLL* right lower lobe, *PM* pulmonary metastasectomy, *VATS* video-assisted thoracic surgery

*Unknown cases were excluded

Solitary lung metastases were present in 22 cases. The morphology was round or oval in 28 cases and other in 7 cases. Ground-glass opacity nodules (GGNs) and cavity lesions were present in four and six cases, respectively. No calcified lesions were observed. The median consolidation-tumor ratio was 1.0 (range, 0.33–1.0). No preoperative histological diagnoses of pulmonary nodules were made.

Preoperative chemotherapy for lung tumors was administered in 12 cases. The median time from detection to PM was 5 (range, 0–35) months. Video-assisted thoracic surgery was the most common approach for PM. Wedge resection, segmentectomy, and lobectomy were performed in 25, 3, and 7 cases, respectively. Factors of pancreatic cancer are presented in Table 2. The stages of pancreatic cancer were I, II, III, and IV in 7, 18, 5, and 3 cases, respectively, all of which had pulmonary metastasis. The histopathological findings of pancreatic cancer were invasive ductal carcinoma, intraductal papillary mucinous neoplasm (IPMN), and unknown in 28, 6, and 1 case, respectively. The median recurrence-free survival after pancreatic cancer surgery was 22 (range, 0–69) months.

**Table 2:** Pancreatic cancer-related factors

Factors	
Stage	
I	7
II	18
III	5
IV	3
Unknown	2
CA19-9 [ng/ml]*	
Median(range)	28.5 (6–1630)
CEA [ng/ml]*	
Median (range)	2.4 (0–15.2)
Localization	
Head	18
Body and tail	16
Unknown	1
Operation	
PD	19
Other	15
Unknown	1
Histological type	
Invasive ductal carcinoma	28
IPMN	6
Unknown	1
Neoadjuvant chemotherapy	
CRT	14
Chemotherapy	2
None	8
Unknown	11
Adjuvant chemotherapy	
( +)	31
(−)	3
Unknown	1
Median relapse-free survival [months]	
Median (range)	22 (0–69)

*CEA* carcinoembryonic antigen; *CA19-9* carbohydrate antigen 19–9, *PD* pancreaticoduodenectomy; *IPMN* intraductal papillary mucinous neoplasm

*Unknown cases were excluded

### Clinical courses

**Fig. 1:**
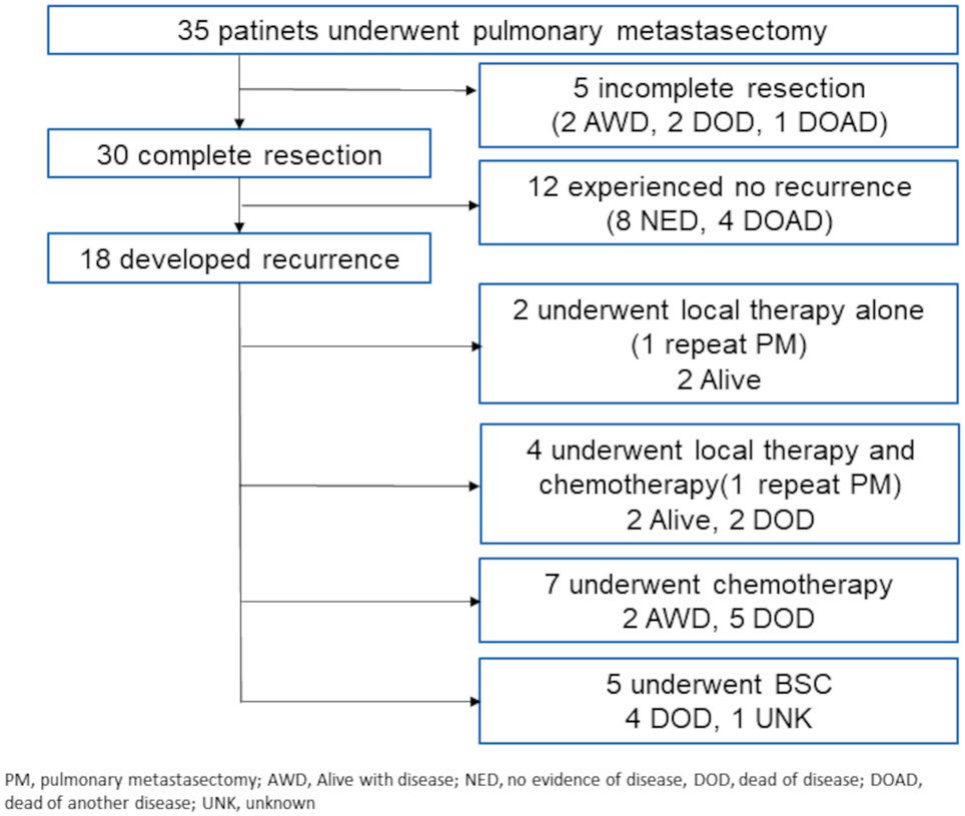
Clinical courses after the first pulmonary metastasectomy. *PM* pulmonary metastasectomy, *AWD* alive with disease, *NED* no evidence of disease, *DOD* dead of disease, *DOAD* dead of another disease, *UNK* unknown

The clinical course of the patients after the first PM procedure is shown in Fig. 1. In five cases, complete resection could not be achieved due to multiple lung metastases. Of the 30 patients who underwent complete resection of lung metastases, 12 did not experience recurrence, while 18 did experience recurrence. Of the 18 patients who experienced recurrence, the sites of the first recurrence after PM were lung/pleura (*n* = 13), local recurrence of the primary tumor (*n* = 1), and other sites (*n* = 4). Of these 18 patients, 6 received local therapy (2 received stereotactic body radiation therapy [SBRT] for lung metastases, 2 received PM, 1 received CyberKnife for brain metastases, and 1 received radiotherapy [RT] for vertebral metastases), and two patients underwent repeat PM once each. Other causes of death, in addition to pancreatic cancer, included cholangitis in one case, interstitial pneumonia in another, and pneumonia in yet another, and the causes remained undetermined in two cases.

### Survival analyses

Survival analyses were subsequently performed on 35 patients who underwent resection of lung metastases. The 3- and 5-year survival rates from pancreatectomy were 88.3 and 64.5%, respectively, whereas the 3- and 5-year survival rates from lung resection were 44.1 and 28.3%, respectively. The median time from recurrence to death was 31.5 months, the time from lung resection to death was 20.0 months, and the time from pancreatic resection to death was 59.0 months. Three cases of a recurrence-free survival of more than five years after pulmonary nodule resection were also observed.

### Predictors of the OS

A univariate analysis of survival-related factors was performed in 35 patients who underwent resection of lung metastases. The results of the univariate analysis of the survival after primary surgery and PM are shown in Table 3. In the univariate analysis of the survival, factors with a DFI of < 15 months were significantly associated with the P-OS (*p* = 0.0007), whereas no factors were significantly associated with the L-OS.

**Table 3:** Results of a univariate analysis

		Overall survival after				
	Pancreatic surgery (P-OS)			Lung surgery (L-OS)		
Variables	HR	95% CI	*P* value	HR	95% CI	*P* value
Age			0.96			0.96
< 71 years	1.00			1.00		
≥ 71 years	1.03	0.40–2.67		0.98	0.38–2.49	
Brinkman Index*			0.62			0.61
0	1.00			1.00		
> 0	1.28	0.48–3.41		1.29	0.49–3.40	
DFI from pancreas surgery			0.0007			0.065
≥ 15 months	1.00			1.00		
< 15 months	6.87	2.24–21.0		2.56	0.94–6.93	
Tumor size			0.27			0.25
< 12 mm	1.00			1.00		
≥ 12 mm	1.75	0.65–4.66		1.79	0.67–4.78	
Distance from pleural surface			0.94			0.83
< 1.0 mm	1.00			1.00		
≥ 1.0 mm	0.97	0.38–2.48		1.11	0.44–2.81	
Number of lung nodule			0.76			0.67
1	1.00			1.00		
> 1	1.16	0.45–3.00		1.23	0.48–3.18	
Side			0.24			0.33
One	1.00			1.00		
Both	1.82	0.67–4.92		1.63	0.61–4.35	
CA19-9 (pre PM)			0.56			0.47
< 19.4 ng/ml	1.00			1.00		
≥ 19.4 ng/ml	1.32	0.51–3.43		1.45	0.54–3.91	
CEA (pre PM)			0.28			0.31
< 4.1 ng/ml	1.00			1.00		
≥ 4.1 ng/ml	1.69	0.65–4.39		1.65	0.63–4.34	
Approach for PM			0.074			0.067
VATS	1.00			1.00		
Thoracotomy	2.82	0.90–8.78		2.86	0.93–8.82	
Operation for PM			0.17			0.17
Others	1.00			1.00		
Lobectomy	2.09	0.72–6.04		2.08	0.73–5.89	
Postoperative hospital stay			0.59			0.65
< 9 days	1.00			1.00		
≥ 9 days	1.30	0.50–3.41		1.25	0.48–3.24	
Induction therapy for PM			0.11			0.35
(−)	1.00			1.00		
( +)	2.31	0.83–6.45		1.59	0.60–4.22	
Chemotherapy after PM			0.38			0.71
(−)	1.00			1.00		
( +)	1.52	0.60–3.87		1.20	0.47–3.08	
Stage*			0.20			0.33
I + II	1.00			1.00		
III + IV	0.44	0.13–1.55		0.54	0.15–1.88	
Operation for pancreas cancer*			0.93			0.91
Others	1.00			1.00		
PD	0.96	0.38–2.44		0.95	0.37–2.41	
Adjuvant chemotherapy*			0.44			0.46
(−)	1.00			1.00		
( +)	2.22	0.29–16.92		2.16	0.28–16.48	
Histological type*			0.72			0.66
Invasive ductal carcinoma	1.00			1.00		
Others	0.79	0.23–2.77		0.76	0.22–2.65	

*CI* confidence interval, *DFI* disease-free interval, *HR* hazard ratio, *PD* pancreaticoduodenectomy, *PM* pulmonary metastasectomy, *VATS* video-assisted thoracic surgery

*Unknown cases were excluded

**Fig. 2:**
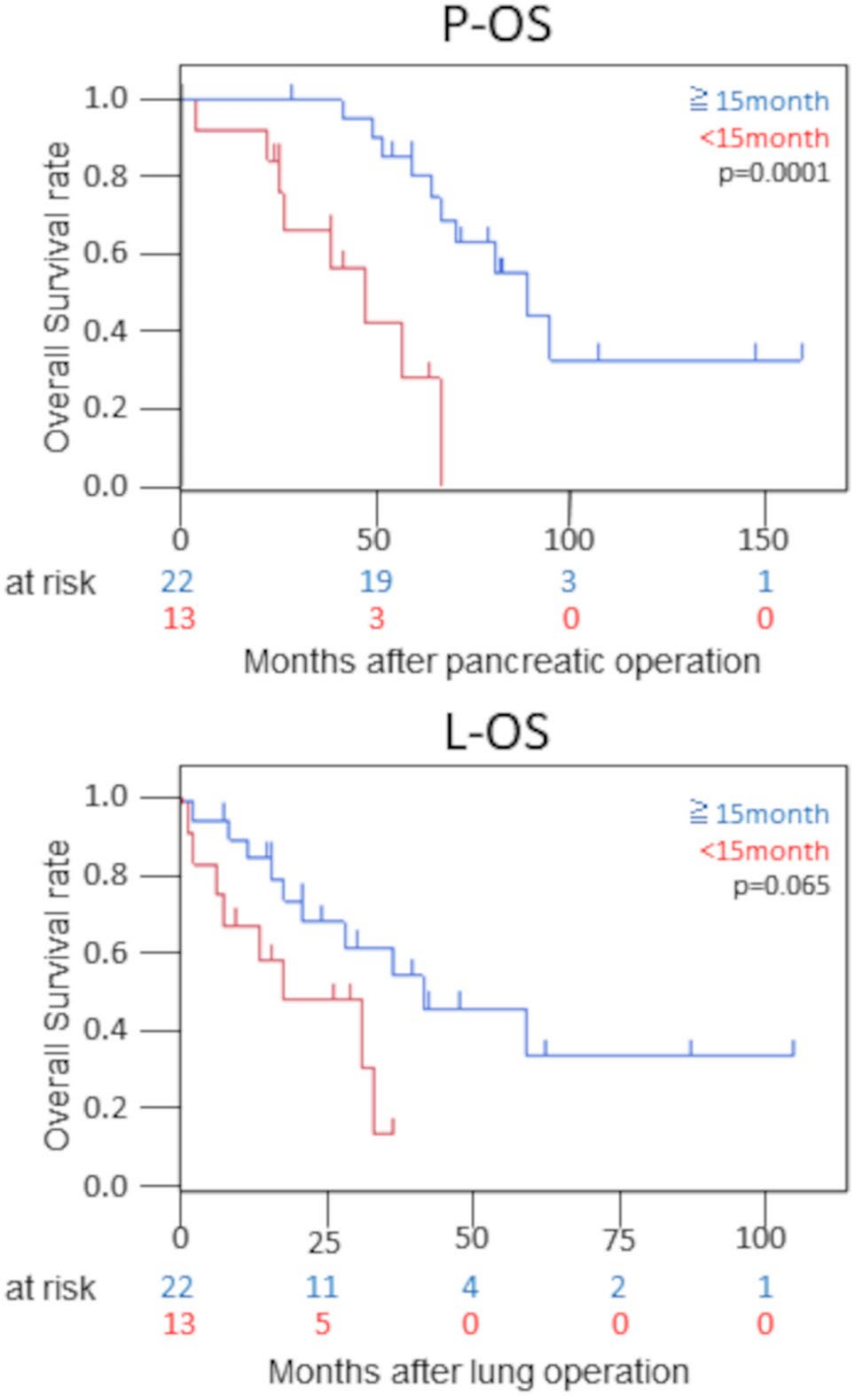
The overall survival according to DFI. **A** The overall survival after pancreatic operation. **B** The overall survival after lung surgery. *DFI* disease-free interval, *P-OS* overall survival after pancreatic resection, *L-OS* overall survival after lung resection

Twenty-two of 35 patients with a DFI of ≥ 15 months had a significantly better P-OS than those with a DFI of < 15 months (*p* = 0.0001), and the 3- and 5-year survival rates were 100 vs. 66.6% and 80.1 vs. 28.6%, respectively, and tended to show a better L-OS than those with a DFI of < 15 months (*p* = 0.065) (Fig. 2). However, among patients with a DFI of < 15 months, 2 patients survived more than 5 years after pancreatic cancer resection. No marked differences in the background characteristics between the patients with a DFI of ≥ 15 and those with a DFI of < 15 months were noted (Table 4).

**Table 4:** Patients’ characteristics

Factor	≥ 15 months	< 15 months	*p* value
Age, years			0.41
Median (range)	71.5 (55–90)	71 (60–81)	
Brinkman Index*			0.26
Median (range)	0 (0–3000)	475 (0–3000)	
Tumor size, mm			0.84
Median (range)	12 (6–21)	14 (6–28)	
Distance from pleural surface, mm			0.9
Median (range)	0.5 (0–35)	2.5 (0–15)	
Number of lung nodule			0.51
Median (range)	1 (1–13)	1 (1–10)	
Side			0.98
One	17	10	
Both	5	3	
CA19-9, ng/ml*			0.75
Median (range)	20 (2–204)	18.0 (0–480.6)	
CEA, ng/ml			0.36
Median (range)	3.85 (1–35)	5.0 (1.8–9.7)	
Approach for PM			0.57
VATS	20	11	
Thoracotomy	2	2	
Operation for PM			0.47
Wedge	17	8	
Segmentectomy	1	2	
Lobectomy	4	3	
Postoperative (PM) hospital stay, days			0.47
Median (range)	9 (4–21)	10 (4–26)	
Induction chemotherapy for PM			0.061
( +)	5	7	
(−)	17	6	
Chemotherapy after PM			0.4
( +)	7	6	
(−)	15	7	
Stage of pancreatic cancer			0.57
I	5	2	
II	9	9	
III	4	1	
IV	2	1	
Unknown	2	0	
Operation for pancreatic cancer			0.11
PD	14	5	
Other	7	8	
Unknown	1	0	
Adjuvant chemotherapy for pancreatic cancer			0.85
( +)	19	12	
(−)	3	1	
Unknown	1	0	
Histological type for pancreatic cancer			0.51
Invasive ductal carcinoma	18	10	
IPMN	3	3	
Unknown	1	0	

*PM* pulmonary metastasectomy, *VATS* video-assisted thoracic surgery

*Unknown cases were excluded

## Discussion

It was reported that patients generally underwent resection of pulmonary metastases after meeting the following criteria: (1) complete resection of the pulmonary metastasis (or metastases) was considered achievable; (2) the metastatic lesions were limited to the lungs or extrapulmonary distant metastases was already controlled or controllable if present; (3) the patient’s primary tumor was already controlled or controllable; (4) lymph node metastasis from the pulmonary lesion was determined to be absent by a preoperative evaluation; (5) the general condition of the patient was good, and the patient’s respiratory function was sufficient to tolerate pulmonary resection [17, 18].

However, the prognosis of pancreatic cancer is poor, and there are not many coherent reports of resection of lung metastases after pancreatic cancer surgery, with a majority covering the period up to 2015. In 2011, Arnaoutakis et al. reported a significant increase in the median OS after relapse (18.6 vs. 7.5 months) in 31 patients with single postoperative lung metastasis from pancreatic cancer between 2001 and 2009, comparing 9 cases of lung resection and 22 cases of non-resection [19]. Subsequent reports have reported a median OS of 28–37.3 months after resection or detection of lung metastases from pancreatic cancer [7, 20, 21]. Conversely, owing to the small number of cases, there are a few reports of risk factors for the OS. Robinson et al. suggested that the recurrence-free interval might influence the OS in patients with resected pulmonary metastases from pancreatic cancer; however, they did not find a statistically significant difference [20]. In 2022, Homma et al. used a multicenter database to examine 32 cases of resection of postoperative lung metastases from pancreatic cancer and performed univariate and multivariate analyses of the first recurrence to death. They reported for the first time that the number of lung metastases and the presence of chemotherapy after lung resection were risk factors for postoperative lung metastases (Table 5) [7]. In contrast, recent advances have been made in postoperative adjuvant chemotherapy for pancreatic cancer. Based on the results of the CONKO-001 trial reported in 2007 [22] and 2013 [23] and the JSAP-02 trial reported in 2009 [24], postoperative adjuvant chemotherapy is recommended for pancreatic cancer that has undergone curative resection. The treatment of pancreatic cancer is changing, and the prognosis is also expected to change.

**Table 5:** Previous reports on pulmonary metastasectomy for pancreatic cancer

Author	Year	Study period	Total number of Patients	Prognosis (months)	Factors associated with the prognosis
Arnaoutakis [23]	2011	1996–2009	9	18.6*	NA
Robinson et al. [24]	2016	1996–2015	16	28**	NA
Okui et al. [25]	2017	2008–2015	6	37.3**	NA
Kurahara et al. [26]	2020	2000–2017	7	36.5*	NA
Homma et al. [10]	2022	2010–2014	32	29.2*	(Multivariate) solitary metastases, postoperative chemotherapy
Present study	2022	2009–2021	35	31.5*, 20**	(Univariate) disease-free interval

*median time from recurrence to death, **median time from lung resection to death

In the present study, the median OS after resection of pancreatic cancer was 59.0 months, and the median OS after PM was 20.0 months, values that are slightly shorter than in previous reports. This may be due to the inclusion of cases with a short observation period. The DFI as a predictor of the OS was highlighted as a possibility in the study by Robinson et al. [20], although that study did not show a significant difference; however, in the present study, the results of the univariate analysis of the P-OS and the Kaplan–Meier curve showed a significant difference.

There have been recent changes in treatment algorithms for pancreatic cancer. In addition to gemcitabine, combination therapies, including FOLFIRINOX and gemcitabine/nab-paclitaxel, are widely used to treat metastatic pancreatic cancer. Although the presence of chemotherapy after primary tumor resection or before or after PM surgery was not a significant risk factor in the present study, it is possible that the longer DFI group had a longer P-OS that the longer DFI group had a longer P-OS than the shorter DFI group, which may have also led to a better prognosis in patients who received more of these treatments than in others. In addition, the group with a DFI of ≥ 15 months tended to have a longer L-OS than the group with a DFI of < 15 months; however, no statistically significant differences were found. This is likely because several of the patients had a short follow-up period; these patients will thus need to be carefully monitored in the future. However, there are cases wherein a long-term survival after PM has been achieved even in patients with a DFI of < 15 months; we therefore believe that surgery may be effective in some patients with lung metastases.

Of note, the study by Homma et al. reported that the number of lung metastases and the presence of chemotherapy after lung resection were risk factors; however, neither of these was found to be a risk factor in the present study. The presence of multiple pulmonary metastases may not have been a risk factor in our study because the indications for surgery vary among institutions. Furthermore, owing to the 10-year study period, the type of chemotherapy performed after pulmonary resection may have varied widely, leading to inconsistent data. Furthermore, tumor markers have been highlighted as risk factors for peritoneal metastasis in colorectal cancer. However, in the present study, high levels of either carcinoembryonic antigen (CEA), carbohydrate antigen19-9 (CA19-9), or both were not found to be risk factors for the OS. In addition, the examination of risk factors showed that patients with pancreatic cancer stages III and IV tended to have a better OS than those with pancreatic cancer stages I and II. Indeed, all three cases in the stage IV group died during the follow-up period, whereas four of the five patients in the stage III group survived during the follow-up period.

Homma et al. revealed that, compared with liver metastasis or peritoneal dissemination, the prognosis is better when the first metastasis of pancreatic cancer is in the lungs [7], suggesting that even in stage III cases, when the first metastasis is in the lungs, lung resection may be expected to improve the prognosis. Conversely, all stage IV patients died during the follow-up period, suggesting that careful consideration should be given to pulmonary resection for stage IV lung metastases.

When a lung nodule is found following pancreatic cancer surgery, differentiating between metastatic and primary lung tumors is often difficult. In the present study, we reviewed imaging findings; however, no consistent findings regarding margins or cavitary lesions were noted. Calcification was not observed in all patients; however, the percentage of calcification in primary lung cancer is reported to be approximately 5.6%–10.6%, and calcification cannot differentiate primary lung cancer from metastatic lung tumor [25, 26]. Metastatic lung tumors are often considered to have well-defined and similarly rounded margins. In this study, it was observed that 80% of the pancreatic metastatic nodules were circular or oval in shape, and the median consolidation-tumor ratio was 1.0. Lung metastases from pancreatic cancer were mostly solid tumors. However, Li et al. reported that, of 59 cases of primary lung cancers, 15 were solid nodules, and 27 were not round or oval in shape [27]. These findings may prove valuable in differentiating primary lung cancer from lung metastases. Of note, some GGN lesions required differentiation from lung cancer, making it difficult to make a preoperative diagnosis based on imaging. Imaging differentiation between pulmonary metastasis of pancreatic cancer and primary lung cancer is therefore an issue for future study.

Several limitations associated with the present study warrant mention. First, the relatively small sample size limited the power of our statistical findings. Second, there may have been differences in patient selection among the institutions. From the thoracic surgeon’s point of view, the indications for PM were consistent with those shown in the *Materials and methods* section above; however, there may have been some selection bias in place before referral to the thoracic surgery departments, as the doctor-in-charge who treated the primary tumor made the judgment. Because thoracic surgeons at each institution determined what procedure to perform for lung metastases, no significant difference between lobectomy and sublobar resection, including segmentectomy or wedge resection, was noted. Which technique should be selected is a subject for further study. Third, this was a retrospective study and not a prospective trial. Fourth, no genetic analysis was performed on individual metastatic tumors. Benign pulmonary nodules and primary lung adenocarcinoma are especially common in pulmonary nodules and may be difficult to distinguish from true lung metastases.

In our study, IPMN and invasive ductal carcinomas were evaluated in conjunction. Among IPMNs, those that manifest lung metastases have been reported to possess a more aggressive phenotype and a poorer prognosis than others, and our univariate analysis also showed no discernible differences in the OS among histological types for pancreatic cancer; still, they are distinct from invasive ductal carcinomas. However, the sample size of this study was limited, including only six cases of IPMN, which was inadequate to consider them as separate entities. Therefore, further research in the form of larger scale studies is necessary in this area. However, only a few studies concerning resection of pulmonary nodules following pancreatic cancer surgery have been this large, making our study significant.

Whether PM is a better treatment than systemic therapy or SBRT remains debatable. In recent years, SBRT has been widely used to manage pulmonary metastases of various cancers, and reasonable local control rates have been achieved [28]. Therefore, the results of this study indicate that PM is beneficial for patients with lung metastases of pancreatic cancer. Nevertheless, further studies will be required to determine if PM is the best treatment for these patients.

However, the presence of long-term survivors, the variety of imaging findings that make a preoperative diagnosis difficult, and the absence of risk factors related to the OS other than DFI suggest that surgery may be considered if the patient’s operative tolerance allows it.

## Conclusions

This retrospective study showed that some patients may benefit from surgical treatment for lung metastases after pancreatic cancer surgery. The DFI influences the treatment of pancreatic cancer and may also affect the survival after metastasectomy. Further studies are required to better select patients who may benefit from pulmonary resection.
